# Non‐Pulmonary Mechanisms of Action of Ambroxol in In Vivo Experimental Models: a Systematic Review

**DOI:** 10.1111/fcp.70077

**Published:** 2026-03-13

**Authors:** Michelline Joana Tenório Albuquerque Madruga Mesquita, Anne Caroline Silva Nogueira da Cruz, Rafael de Abreu Lima, Joana Tenório‐Meireles, Arney José Nogueira Farias, Isabela Nogueira Santos, Gustavo Frota, Taciana Gabrielle Pinheiro de Moura Carvalho, Rafael Antônio Freire Carvalho, Jorge Antônio Meireles‐Teixeira, Tereza Prazeres, Rafael Cardoso Carvalho, Maria do Socorro de Sousa Cartágenes, João Batista Santos Garcia

**Affiliations:** ^1^ Medicine Department I Federal University of Maranhao (UFMA) and Post‐Graduate Program in Health Sciences (UFMA) Sao Luis Maranhao Brazil; ^2^ Post‐Graduate Program in Health Sciences Federal University of Maranhao Sao Luis Maranhao Brazil; ^3^ Nursing Department, Federal University of Maranhao, and Multiprofessional Residence Program in Health University Hospital of Maranhao (HUUFMA) Sao Luis Maranhao Brazil; ^4^ University CEUMA Sao Luis Maranhao Brazil; ^5^ Federal University of Maranhao Sao Luis Maranhao Brazil; ^6^ Graduate Program in Biotechnology – RENORBIO Federal University of Maranhao Sao Luis Maranhao Brazil; ^7^ Medicine Departament I Federal University of Maranhao (UFMA) Sao Luis Maranhao Brazil; ^8^ Medical School – Pinheiro Campus Federal University of Maranhao (UFMA) Pinheiro Maranhao Brazil; ^9^ Department of Pathology, Center for Biological and Health Sciences Federal University of Maranhao Sao Luis Maranhao Brazil; ^10^ Physiological Sciences Department Federal University of Maranhao Sao Luis Maranhao Brazil; ^11^ Medicine Departament II Federal University of Maranhao Sao Luis Maranhao Brazil

**Keywords:** ambroxol, analgesic, anti‐inflammatory agent, antioxidant, drug repositioning, in vivo

## Abstract

**Background:**

Repositioning offers a cost‐effective approach to discovering new therapeutic applications for existing medications. Ambroxol, primarily used as a mucolytic for respiratory diseases, has demonstrated anti‐inflammatory, analgesic, and antioxidant properties, suggesting potential benefits in non‐pulmonary conditions. This study conducted a systematic review to evaluate the efficacy of ambroxol in experimental disease models unrelated to the respiratory system.

**Methods:**

Following registration in the Open Science Framework and adherence to the PICO strategy for formulating the guiding question, searches were performed in PUBMED/MEDLINE, EMBASE, and SCOPUS using the keywords: (Ambroxol) AND (Anti‐Inflammatory Agents OR Analgesics OR Antioxidants) AND (Animals OR in vivo). The SYRCLE tool assessed methodological quality. Among 353 identified records, eight articles met eligibility criteria.

**Results:**

These studies investigated ambroxol's effects in models of gastric lesions, neuropathic pain, psoriasis‐like skin inflammation, hemorrhagic cystitis, and ischemia/reperfusion injuries in the liver and kidneys. Ambroxol doses ranged from 5 to 1000 mg/kg, predominantly administered orally. Its antioxidant properties were demonstrated by reducing free radicals and increasing enzymatic activity (SOD, CAT, GSH). Anti‐inflammatory effects included a decrease in pro‐inflammatory cytokines (TNF‐α, IL‐1β) and histological improvements. Antinociceptive action was observed through inhibition of voltage‐gated sodium channels and reduction of oxidative stress, alleviating neuropathic pain.

**Conclusions:**

Despite ambroxol's widespread clinical use, limited research has explored its non‐respiratory applications. Existing studies suggest its promising therapeutic potential, reinforcing the need for further investigation into its role as an alternative treatment for various inflammatory and oxidative stress–related conditions beyond pulmonary diseases.

**Abbreviations**
ABX: ambroxol
ACH: acetilcholine
AED: animal equivalent dose
ALT: alanine aminotransferase
AST: aspartate aminotransferase
ALP: alkaline phosphatase
BUN: blood urea nitrogen
CAT: catalase
CR: creatinine
CYP: cyclophosphamide
GSH: glutathione
IL: interleukin
IL‐1β: interleukin‐1 beta
IL‐6: interleukin‐6
IL‐10: interleukin‐10
IL‐17: interleukin‐17
IL‐22: interleukin‐22
IL‐23: interleukin‐23
IMQ: imiquimod
IND: indomethacine
LDH: lactate dehydrogenase
LPS: lipopolysaccharide
MDA: malondialdehyde
MPO: myeloperoxidase
NF‐κB: nuclear factor kappa B
OSF: Open Science Framework
PRG: pregabalin
QCRI: Qatar Computing Research Institute
SC: subcutaneous
SOD: superoxide dismutase
SYRCLE: Systematic Review Center for Laboratory Animal Experimentation
TBARS: thiobarbituric acid reactive substances
TGF‐β: transforming growth factor beta
TLR: Toll‐like receptors
TNF‐α: tumor necrosis factor alpha

## 1 Introduction

Drug repositioning has emerged as a promising alternative in response to the challenges of developing new medications, as it seeks to identify new applications for a drug beyond its originally approved indication. This approach provides a solution to the lengthy, costly, and highly uncertain process of drug development, in which only 2.01% of innovations in chemical molecules reach the market [1].

The significant reduction in time and costs associated with drug repositioning is due to the already well‐known safety and pharmacokinetic profile of these molecules, facilitating the rapid advancement of research for evaluation in clinical models and Phase II and III trials. This technique expands the therapeutic potential of previously approved drugs [2].

Ambroxol (ABX) or trans‐4‐(2‐amino‐3,5‐dibromobenzylamino)‐cyclohexanol is a chemical compound classified as a mucolytic agent, widely used for decades in the treatment of respiratory diseases due to its ability to stimulate mucociliary clearance, improve mucus viscosity, and facilitate expectoration [3].

It is an active metabolite of bromhexine, isolated from the plant species *Adhatoda vasica* . ABX is clinically used for the treatment of respiratory diseases associated with viscous or excessive mucus in chronic bronchitis. Additionally, studies have shown that ABX possesses anti‐inflammatory, analgesic, antiviral, antibacterial, antifungal, and antifibrotic properties through various mechanistic actions, including antioxidant potential by scavenging free radicals and reducing the release of histamine, human leukotrienes, mast cells, and leukocytes [4].

ABX is already widely used in clinical practice, demonstrating a safe use profile and well‐established pharmacodynamics. Recent investigations have revealed additional properties, expanding its therapeutic potential beyond the respiratory tract. Despite these perspectives, knowledge about the action and efficacy of ABX in experimental models outside the respiratory context remains limited. Therefore, gathering and systematically organizing evidence is essential to support future therapeutic applications of this drug.

Therefore, the present study aimed to conduct a systematic literature review to evaluate the anti‐inflammatory, analgesic, and antioxidant properties of ABX in in vivo experimental models of non‐respiratory diseases.

## 2 Methods

This is a systematic review study, registered in the Open Science Framework (OSF) under DOI number 10.17605/OSF.IO/ZXTVR and based on the methodological guidelines for conducting systematic reviews and meta‐analyses of randomized clinical trials [5]. The research was guided by the following key question: Does ABX demonstrate efficacy when used as an anti‐inflammatory, analgesic, and antioxidant agent in experimental models of diseases beyond the respiratory tract? Subsequently, it was structured according to the PICO strategy (Population, Intervention, Comparison, and Outcomes), where the characteristics of the population (rats with inflammatory conditions), intervention (effect of ABX administered at different doses), comparison (other medications used, results before and after ABX administration), and outcome (anti‐inflammatory, analgesic, and/or antioxidant action) were combined (Table 1).

**TABLE 1:** PICO strategy.

Studied domain: use of ambroxol	
Population	Rodents in inflammatory conditions
Intervention	Effect of ambroxol administered at different doses
Comparison	Other medications used, results before and after ambroxol
Outcome	Anti‐inflammatory, analgesic, and/or antioxidant action

### 2.1 Literature Search

The search was conducted in the PubMed/MEDLINE, Embase, and Scopus databases. It was carried out between September and November 2024, with no restrictions on year or language for the selected studies. For the search, Boolean operators "AND" and "OR" were used in combination with controlled descriptors, according to the following search strategy: (Ambroxol) AND (Anti‐Inflammatory Agents OR Analgesics OR Antioxidants) AND (Animals OR in vivo).

The studies retrieved from all combinations and databases were selected using a free web‐based review platform called Rayyan, developed by the Qatar Computing Research Institute (Rayyan QCRI) [6]. The selection process involved duplicate removal, identification, selection, and eligibility assessment.

### 2.2 Inclusion Criteria

Initially, studies that involved animal experimentation and the use of ABX in analgesic, anti‐inflammatory, and antioxidant models for non‐pulmonary diseases were included.

### 2.3 Exclusion Criteria

Articles that were not original, that addressed in vitro or human experimental models, that focused on the pulmonary effect of ABX, or that investigated neurodegenerative diseases and chaperone mechanisms were excluded.

### 2.4 Study Selection

The study selection process was conducted independently by two blinded reviewers (Michelline Joana Tenório Albuquerque Madruga Mesquita and João Batista Santos Garcia), and disagreements were resolved by a third reviewer. In the first stage, the titles of the references identified through the search strategy were read, and potentially eligible studies were preselected. In the second stage, the titles and abstracts were reviewed, and the articles chosen in this step were analyzed in full for inclusion or exclusion in the review.

### 2.5 Data Extraction

The data from the included studies were extracted by one evaluator (M. J. T. A. M. M). For this purpose, a form adapted from the Cochrane Development, Psychosocial and Learning Problems (Cochrane) (https://dplp.cochrane.org/data‐extraction‐forms) was used, and the data were recorded in a Microsoft Excel spreadsheet. The extracted variables included study characteristics (author/year of publication, country of study), sample characteristics (sample size, animal characteristics—species, sex, weight), study objective, type of inflammatory model used, intervention (dose, route of administration, and treatment duration), evaluation parameters, results, and main conclusions.

### 2.6 Interspecies Dose Adjustment

To account for interspecies differences in metabolic rates between rats and mice, the methodology for calculating the animal equivalent dose (AED) was adopted, based on the body surface area normalization method [7].

Accordingly, estimated average body weights were used for rats (220 g) and mice (32 g), considering the strain standards reported in the studies. Based on this, the minimum and maximum doses administered in mice were converted into their rat‐equivalent doses to facilitate a more accurate interpretation of pharmacological exposure and enhance the consistency of the findings.

### 2.7 Methodological Quality Assessment

To assess the methodological quality of the animal studies included in this review, the Systematic Review Center for Laboratory Animal Experimentation (SYRCLE) tool was used to evaluate the risk of bias in animal studies. This tool includes the following categories: selection bias, performance bias, detection bias, attrition bias, reporting bias, and other sources of bias. Ten questions were applied to the articles included in the systematic review, with possible answers being “YES,” indicating a low risk of bias (when the methodological criterion was clearly met); “NO,” indicating a high risk of bias (when the criterion was not met or was inadequately applied); and “UNCLEAR” (when the information was not available or was insufficient for a proper judgment) [8].

Because this study involved the analysis of existing published research, it did not require ethical approval. The data used were publicly available and did not involve any direct interaction with animals, humans, or their personal information. Furthermore, no authors were contacted to obtain additional information about their publications, as the analysis focused solely on the data within the publications themselves.

## 3 Results

**FIGURE 1:**
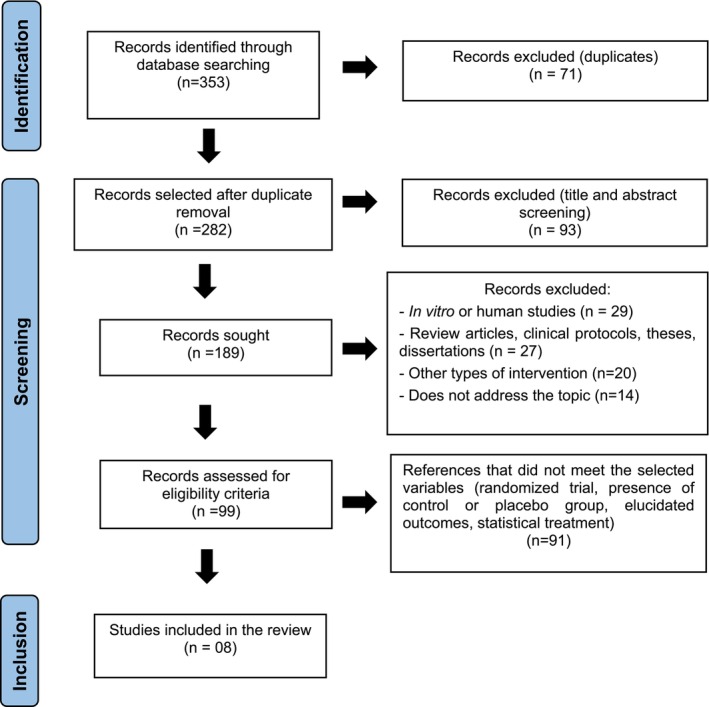
Identification of studies through databases according to PRISMA recommendations.

A total of 353 articles were identified in the electronic databases using the search strategy. During the selection process, 71 duplicate references were removed, resulting in 282 articles. The titles and abstracts of these articles were screened, leading to the exclusion of 93 references, leaving a total of 189 studies. Regarding study type, 29 studies were excluded for being conducted in humans or in vitro; 27 were clinical protocols, review articles, theses, or dissertations; 21 articles investigated ABX for respiratory tract diseases; and 14 studies did not address the relevant topic. This process resulted in 99 articles, which were subjected to independent screening using the Rayyan software. Screening was performed by two reviewers, and in case of a tie, a third author provided an opinion. Consequently, the full texts of the 99 records were analyzed regarding the methodology used, results, and conclusions. After this stage, references that did not meet the selected variables for this review (randomized trial, presence of a control or placebo group, clearly defined outcomes, statistical analysis) were excluded, resulting in a final sample of eight studies (Figure 1).

### 3.1 Characteristics of Included Studies

In this review, eight studies were included. The average number of animals evaluated was 37, ranging from 6 to 108, with two studies not reporting the number of participants. Most of the subjects were rats (*n* = 14), predominantly male (*n* = 36), from the Wistar strain (*n* = 8). The research objectives varied, but all explored specific aspects of ABX efficacy in inflammatory, painful, or oxidative conditions.

In five studies (62.5%), the anti‐inflammatory effect was evaluated using experimental models such as neuropathy, hepatic ischemia/reperfusion, psoriasis, hemorrhagic cystitis, and renal ischemia/reperfusion. Among these models, the neuropathic pain model was the most prevalent, representing 37.5% of the evaluated studies. Additionally, 50% of the studies investigated the antioxidant activity of the drug, while 37.5% explored its analgesic effect.

Considering the dose conversion using the AED calculation based on studies conducted with mice, it was possible to estimate an adjusted dose range corresponding to rats. Thus, in models evaluating anti‐inflammatory activity, ABX doses varied widely, from 5 to 1000 mg/kg, administered through different routes, including oral, intraperitoneal, subcutaneous, and topical. For antioxidant activity, the adjusted doses ranged from 5 to 140 mg/kg, via oral, intravenous, subcutaneous, and intraperitoneal routes. Regarding analgesic effects, only studies conducted in rats were found, with doses ranging from 30 to 1000 mg/kg, always administered orally, with one study evaluating the combination of ABX with other agents, such as pregabalin.

The treatment duration with ABX ranged from 6 h to 4 weeks, depending on the experimental model. Antioxidant and anti‐inflammatory studies generally adopted shorter protocols, ranging from 6 h to 7 days, whereas neuropathic pain and analgesia models involved longer treatments, lasting from 5 days to 4 weeks. In studies with therapeutic combinations, such as ABX and pregabalin, the duration reached up to 21 days (Table 2).

**TABLE 2:** Characterization of animal model studies included in this review.

Author/year	Country	Study population	Objective	Research target/intervention model	Dose and administration route	Treatment duration
Štětinová et al. (2004) [9]	Czech Republic	Female Wistar rats (*n* = NR*) 200–250 g	Evaluate the antioxidant action of ambroxol (ABX)	Indomethacin (IND)‐induced gastric lesion	(1) Body lesions: ABX 10, 30, and 50 mg/kg (oral); (2) Antral lesions: 50 mg/kg (oral)	Pre‐induction: (1) Body lesions: 5, 30, 60 min before IND; (2) Antral lesions: 30 min before IND and 1 h after feeding/6 h
Gaida et al. (2005) [10]	Germany	Male Wistar and Sprague–Dawley rats (*n* = NR*) 200–350 g	Investigate the effect of ABX on chronic neuropathic and inflammatory pain	Pain: (1) Partial sciatic nerve ligation; (2) Chronic sciatic nerve constriction injury. Inflammation: Freund's adjuvant paw injection	ABX 30, 100, 300, and 1000 mg/kg (oral)	(1) Analgesia: 8–10 days; (2) Inflammation: 5 days
Hama et al. (2010) [11]	United States	Male Sprague–Dawley rats (*n* = 6) 100–150 g	Efficacy of ABX in neuropathic pain control	Surgical clip–induced spinal cord injury	ABX 100, 300, and 1000 mg/kg (oral)	4 weeks
Jiang et al. (2013) [12]	China	Male Wistar rats (*n* = 30) 200–280 g	Evaluate the antioxidant and anti‐inflammatory actions of ABX	Hepatic ischemia/reperfusion injury	ABX 20, 80, and 140 mg/kg (intravenous)	6 h
Bhardwaj et al. (2016) [13]	India	NR*/Sprague–Dawley rats (*n* = 30) 180–250 g	Investigate the neuroprotective and antinociceptive effects of ABX in neuropathic pain	Induction by oxaliplatin injection	(1) ABX 1000 mg/kg (oral); (2) ABX 1000 mg/kg + pregabalin (PRG) 10 mg/kg (oral)	21 days
Sunkari et al. (2019) [14]	India	Male BALB/c mice (*n* = 30) 25‐30 g	Evaluate the anti‐inflammatory and antioxidant effects of ABX	Imiquimod (IMQ) model for psoriasis‐like cutaneous inflammation	ABX 10 and 30 mg/kg (topical) and ABX 2 mg/kg subcutaneous (SC)	7 days
Barut et al. (2019) [15]	Turkey	Male BALB/c Mice (*n* = 108) 25–40 g	Potential anti‐inflammatory and antioxidant effects of ABX	Cyclophosphamide (CYP)‐induced hemorrhagic cystitis	ABX 30, 70, and 100 mg/kg (intraperitoneal)	4 days
Gültekin et al. (2022) [4]	Cyprus and Turkey	Female and male Wistar rats (*n* = 18) 200–250 g	Evaluate the anti‐inflammatory effect of ABX	Renal ischemia/reperfusion injury model	ABX 30 mg/kg (oral)	6 h

Abbreviation: NR, not reported.

The included studies utilized various evaluation parameters to investigate the effects of ABX. Histological analyses were performed in 62.5% of the studies, highlighting changes in tissues such as the stomach, liver, skin, bladder, and kidneys. Biochemical parameters were employed in 62.5% of the studies, including inflammatory markers tumor necrosis factor (TNF‐α), interleukin 1β (IL‐1β), and antioxidant markers such as glutathione (GSH), superoxide dismutase (SOD), catalase (CAT), and malondialdehyde (MDA). Functional and behavioral parameters were assessed in 50% of the studies, covering bladder contractility, thermal and mechanical allodynia, and withdrawal latency in acute and chronic pain models. These parameters were essential for assessing the antioxidant, anti‐inflammatory, and analgesic effects of ABX.

The review demonstrated that ABX exhibits significant anti‐inflammatory, antioxidant, and analgesic effects. A total of 62.5% of the studies reported a significant reduction in inflammatory markers, such as TNF‐α, IL‐1β, and IL‐6, as well as improvements in inflammatory conditions, including psoriasis, hemorrhagic cystitis, and ischemic injuries. The same percentage of studies showed an increase in antioxidant enzyme activity, such as GSH, SOD, and CAT, along with a reduction in oxidative stress markers, including MDA.

Regarding pain control, 37.5% of the studies demonstrated that ABX significantly reduced hyperalgesia and allodynia in a dose‐dependent manner, with effects comparable with those of standard therapies. Histological models indicated that ABX attenuated tissue damage, reduced edema, and prevented structural damage in organs such as the liver, kidneys, and skin.

It was concluded that ABX exhibited dose‐dependent analgesic and antinociceptive properties, leading to a reduction in hepatic, renal, and cutaneous damage, with anti‐inflammatory and antioxidant effects. Additionally, it improved neuropathic pain and bladder contractile function by blocking sodium channels and regulating redox balance (Table 3).

**TABLE 3:** Main findings and statistical significance in the studies included in this review.

Author/year	Evaluation parameters	Results	Key conclusions
Štětinová et al. (2004) [9]	Histological examination of the stomach: (1) Macroscopic evaluation of gastric lesions and (2) Microscopic examination.	(1) Macroscopic: Dose‐dependent inhibition: Best results with ABX 50 mg/kg at 30 min pre‐induction with indomethacin. Antral lesions: 50 mg/kg reduced lesion count but had a larger extension than the vehicle. (2) Microscopic: ABX reduced lesion depth and severity at 50 mg/kg, but antral lesions were not well controlled, forming true ulcers.	Low antioxidant effect despite free radical elimination.
Gaida et al. (2005) [10]	(1) Acute pain model (tail‐flick and hot plate); (2) Chronic pain model (formalin paw test); (3) Neuropathic pain model.	(1) Acute pain model: ABX 1000 mg/kg increased withdrawal latency; (2) Chronic pain model: ABX 1000 mg/kg reduced paw flinching in acute and late phases; (3) Neuropathic pain model: ABX 100 and 1000 mg/kg reduced mechanical allodynia, fully restoring it at the highest dose.	ABX exhibited significant dose‐dependent analgesic properties.
Hama et al. (2010) [11]	(1) Mechanical hypersensitivity model (Von Frey test); (2) Heat hypersensitivity model (infrared test); (3) Rotarod test; (4) Basso, Beattie, Bresnahan locomotor rating scale.	(1) ABX 1000 mg/kg reversed mechanical hypersensitivity by 72% in 60 min; (2) Partial reversal of heat hypersensitivity was observed at 90 min with 100 mg/kg (65%) and 300 mg/kg (61%); (3) ABX did not alter rotarod performance; (4) ABX did not significantly change BBB scores.	ABX exhibited significant antinociceptive effects without impairing coordinated locomotion, suggesting that its mechanism of action involves sodium channel blockade in primary afferent nociceptors.
Jiang et al. (2013) [12]	(1) Histological analysis; (2) Biochemical analysis alanine aminotransferase (ALT) and aspartate aminotransferase (AST); (3) Antioxidant enzyme activity (SOD, CAT, GSH, MDA in liver tissue); (4) Western blot (caspase‐3, Bcl‐2, Bax, JNK).	(1) Histological analysis: ABX 20, 80, 140 mg/kg reduced cytoplasmic discoloration and nuclear condensation, with the best results at 80 and 140 mg/kg; (2) Biochemical analysis: ABX 20, 80, and 140 mg/kg significantly reduced ALT and AST levels; (3) Antioxidant enzyme activity: SOD and CAT significantly increased with ABX 80 and 140 mg/kg; GSH increased and MDA decreased with ABX 20, 80, and 140 mg/kg; (4) Western blot: ABX 140 mg/kg significantly increased Bcl‐2 and reduced JNK; ABX 80 and 140 mg/kg reduced Bax and caspase‐3 levels.	ABX significantly reduced liver damage caused by ischemia/reperfusion injury, increasing antioxidant and anti‐apoptotic activity.
Bhardwaj et al. (2016) [13]	(1) Thermal paw hyperalgesia (hot plate test); (2) Cold paw allodynia (acetone drop method); (3) Cold tail hyperalgesia (cold tail immersion test); (4) Biochemical analysis (superoxide anion generation, total protein, thiobarbituric acid reactive substances (TBARS), tumor necrosis factor‐alpha (TNF‐α), myeloperoxidase (MPO) activity).	(1) ABX 1000 mg/kg and ABX + PRG significantly attenuated thermal paw hyperalgesia, cold tail hyperalgesia, and cold paw allodynia; (2) ABX 1000 mg/kg and ABX + PRG reduced oxidative stress markers (superoxide anion generation, TBARS) and inflammatory mediators (TNF‐α and MPO).	ABX 1000 mg/kg and ABX + PRG significantly improved neuropathic pain, reducing oxidative stress and inflammatory mediators. The antinociceptive and neuroprotective effects of ABX are attributed to voltage‐gated sodium channel blockade.
Sunkari et al. (2019) [14]	(1) Spleen index relative to body weight; (2) Biochemical parameters in skin tissue samples—GSH; (3) Estimation of nitrite levels induced by lipopolysaccharide (LPS) and imiquimod (IMQ); (4) Estimation of SOD; (5) Histological evaluation; (6) Cytokine assessment: IL‐1β, IL‐6, IL‐17, IL‐22, IL‐23, TNF‐α, and transforming growth factor beta (TGF‐β).	(1) ABX 10 and 30 mg/kg (topical) and 2 mg/kg (SC) reduced epidermal hyperplasia, skin thickness, and spleen enlargement, reducing spleen index; (2) ABX 10 and 30 mg/kg (topical) significantly increased antioxidant markers GSH and SOD and reduced nitrite levels; (3) ABX 10 and 30 mg/kg (topical) reduced IL‐1β, IL‐6, IL‐17, IL‐22, IL‐23, TGF‐β, and TNF‐α and increased IL‐10 levels; (4) ABX 10 and 30 mg/kg (topical) significantly attenuated psoriasiform lesions in the histological analysis.	ABX significantly reduced skin inflammation, skin thickness, and splenomegaly. It decreased pro‐inflammatory cytokines and modulated the oxidative/antioxidant redox balance.
Barut et al. (2019) [15]	(1) Bladder contractility studies; (2) Biochemical analysis: MDA; total GSH; TNF‐α; (3) Histopathological analysis.	(1) Pre‐treatment with ABX did not reduce bladder weight increase caused by cyclophosphamide (CYP); (2) ABX 100 mg/kg pre‐treatment significantly increased acetylcholine (ACh) responsiveness; (3) ABX 70 and 100 mg/kg pre‐treatment reduced MDA lipid peroxidation; (4) ABX 30 mg/kg significantly prevented GSH reduction; (5) ABX 30, 70, and 100 mg/kg pre‐treatment prevented TNF‐α level increase; (6) ABX 70 mg/kg significantly reduced mucosal hemorrhage.	ABX improved bladder contractile function, reduced mucosal hemorrhage, and demonstrated antioxidant and anti‐inflammatory effects by decreasing MDA levels and TNF‐α levels while increasing GSH levels.
Gültekin et al. (2022) [4]	(1) Biochemical analysis: alkaline phosphatase (ALP), lactate dehydrogenase (LDH), blood urea nitrogen (BUN), creatinine (CR) levels, and TNF‐α and IL‐1β concentrations; (2) Histopathological analysis.	(1) ABX reduced ALP and LDH levels; (2) ABX significantly decreased BUN and CR levels; (3) ABX significantly reduced TNF‐α and IL‐1β levels; (4) ABX reduced interstitial edema and tubular cell desquamation.	ABX protected renal structures and functions, minimizing cellular and inflammatory damage by reducing ALP, LDH, BUN, CR, TNF‐α, and IL‐1β levels.

Abbreviation: NR, not reported.

### 3.2 Methodological Quality Assessment

The evaluation of the studies using the SYRCLE tool criteria indicated a low risk of bias in animal allocation sequence (75%), baseline characteristics (100%), and random housing (62.5%), suggesting that experimental groups were homogeneous before interventions.

However, allocation concealment showed an uncertain risk in 75% of the studies, and blinding, in both outcome assessment and interventions, presented an uncertain risk in 87.5%, highlighting weaknesses in preventing biases related to knowledge of interventions by assessors.

**FIGURE 2:**
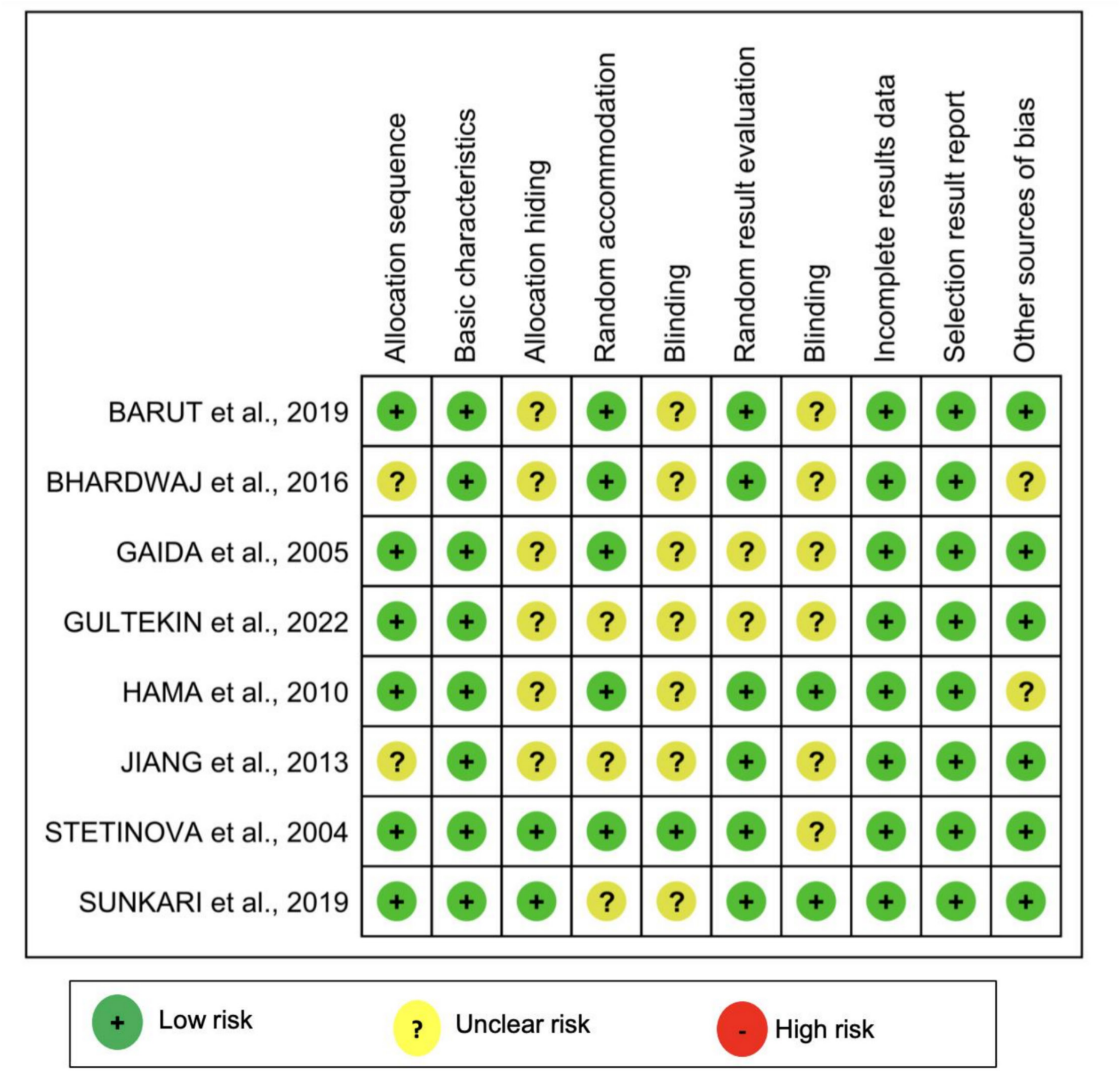
Risk of bias in animal studies assessed by the SYRCLE tool.

Despite these concerns, incomplete outcome data and selective outcome reporting were considered low risk in 100% of the studies, ensuring transparency and methodological reliability in the evaluated studies (Figure 2).

## 4 Discussion

In this systematic review, the potential of ABX as an anti‐inflammatory, antioxidant, and analgesic agent was evaluated in experimental models of diseases unrelated to the respiratory system. The included studies suggest that the drug exhibits therapeutic versatility, with significant effects in various pathophysiological contexts. Recognizing the interspecies heterogeneity between the models used (rats and mice) and the potential metabolic implications for ABX's pharmacokinetics, the AED was calculated to convert the doses administered in mice to adjusted values for rats. This partial standardization aimed to minimize the limitations in dose comparability between species and was incorporated into the analysis of results, allowing for a more robust interpretation of the magnitude and consistency of the observed effects.

### 4.1 Antioxidant Activity

In the study by Jiang et al. in 2013 [12], the dose‐dependent antioxidant effect of ABX was evaluated in a model of ischemia/reperfusion‐induced hepatic injury. The authors used histological parameters and biochemical assays to assess the markers. It was observed that animals receiving higher doses of ABX (80 and 140 mg/kg) exhibited better outcomes in controlling liver injury, with reduced histological damage and decreased serum levels of alanine aminotransferase (ALT) and aspartate aminotransferase (AST). This finding is supported by the study of Stetinova et al. in 2004 [9], which demonstrated a dose‐dependent and time‐related antioxidant effect of ABX in a model of indomethacin‐induced gastric injury. The study reported better modulation of these injuries in both number and extent when ABX was administered 30 min before indomethacin at the highest tested dose (50 mg/kg). These results suggest that the observed benefits may be associated with the antioxidant and anti‐inflammatory properties of ABX, particularly at high doses.

However, when compared with other studies, the in vivo effect of ABX was considered low in the study by Stetinova et al. in 2004 [9], despite the fact that the compound appears to be capable of scavenging a broad spectrum of radicals, which play a key role in the pathogenesis of indomethacin‐induced gastric mucosal injury [16].

The study by Barut et al. in 2019 [15] demonstrated the ability of ABX to control oxidative damage in a model of cyclophosphamide (CYP)‐induced hemorrhagic cystitis. Pre‐treatment with ABX reduced MDA lipid peroxidation and prevented the depletion of GSH. Similar findings were reported in the study by Sunkari et al. in 2019 [14], which evaluated the effect of ABX in an imiquimod (IMQ)‐induced model of psoriasis‐like skin inflammation in rats, observing increased levels of antioxidant markers such as GSH and SOD, as well as reduced nitrite levels. These results were also observed in the study by Jiang et al. in 2013 [12], which reported increased activities of SOD, CAT, and GSH. These findings suggest that ABX exerts its antioxidant action both through the direct modulation of antioxidant enzymes and by preserving components of the cellular antioxidant defense system [17, 18].

### 4.2 Anti‐Inflammatory Activity

In the study by Sunkari et al. in 2019 [14], the positive effect of ABX was evaluated in a psoriasis‐like model induced by the inflammatory agent IMQ. This inflammation model was developed by Van Der Fits and consists of the topical application of IMQ cream, which is commercially available in India, to the shaved dorsal skin region. IMQ is a ligand for Toll‐like receptors (TLR7 and TLR8) and acts as a potent immune activator. It is commonly used for the topical treatment of genital warts caused by the human papillomavirus and other cancerous skin abnormalities [19].

When analyzing the histology of IMQ‐induced skin lesions, the authors observed that topical application of ABX significantly reduced epidermal hyperplasia. Regarding biochemical and immunological parameters, the IMQ group exhibited lower levels of GSH peroxidase and SOD but a higher level of nitrite production compared with the control rats. However, after treatment with ABX at doses of 10 and 30 mg/kg topical and 2 mg/kg subcutaneous, the antioxidant markers GSH and SOD were significantly increased compared with the IMQ control group. Additionally, a reduction in nitrite levels in the skin tissue was also observed [14].

Topical ABX at doses of 10 and 30 mg/kg also effectively counteracted oxidative stress. The drug additionally attenuated elevated cytokine levels in a dose‐dependent manner. Thus, ABX intervention led to a significant reduction in nitrosative and oxidative stress and decreased the levels of cytokines IL‐1β, IL‐6, IL‐17, IL‐22, IL‐23, TGF‐β, and TNF‐α, while simultaneously increasing the expression of the anti‐inflammatory cytokine IL‐10 [14].

Another study evaluated the effects of ABX on pro‐inflammatory cytokines in a rat model of ischemia/reperfusion‐induced renal injury [4]. When assessing biochemical parameters through serum levels of alkaline phosphatase (ALP) and lactate dehydrogenase (LDH), a significant increase in these enzymes was detected in the ischemic injury group without treatment. In contrast, in the group treated with ABX hydrochloride, ALP and LDH activities were significantly reduced. Regarding pro‐inflammatory markers, TNF‐α and IL‐1β levels were analyzed in both groups: the control group (with injury and no treatment) and the ABX‐treated group (with injury and treated with ABX). The findings showed that untreated rats secreted significantly higher levels of TNF‐α and IL‐1β (*p* < 0.0001), whereas these levels were reduced in the ABX‐treated group (*p* < 0.05). Additionally, interstitial edema and tubular cell desquamation were decreased in the group treated with ABX.

A different study also analyzed the protective effect of ABX against oxidative/inflammatory damage, but in this case, induced by CYP in the urinary bladder of BALB/c mice [15]. The study's main findings highlighted a reduction in bladder injury, as ABX treatment significantly decreased mucosal hemorrhage and improved bladder contractile function. Additionally, improvements in biochemical parameters were observed, including cytokine levels and inflammation markers such as total GSH and TNF‐α.

Accordingly, Barut et al. in 2019 [15] observed that CYP treatment significantly increased TNF‐α levels compared with the control group. Additionally, it was found that ABX treatment, at all doses, reduced TNF‐α levels, similar to the group treated with the reference drug. In the study by Su et al. in 2004 [20], which evaluated a murine model of acute lung injury, ABX treatment also reduced TNF‐α levels in bronchoalveolar lavage fluid due to its anti‐inflammatory potential. Similar results were observed in the studies by Malerba et al. in 2008 [17] and Beeh et al. in 2008 [21], which reported the anti‐inflammatory effect of ABX by reducing TNF‐α levels and neutrophil infiltration in the lungs of rats, as well as decreasing the release or production of cytokines such as IL‐1β.

Although the anti‐inflammatory mechanism of ABX is not yet well elucidated for other comorbidities, Ferretti et al. in 1992 [22] suggest that ABX may inhibit phosphodiesterase enzymes, which block the production of pro‐inflammatory cytokines. Additionally, ABX may also inhibit the activation of the pro‐inflammatory transcription factor nuclear factor kappa B (NF‐κB) [21].

### 4.3 Antinociceptive Effect

A study conducted in India investigated the neuroprotective and antinociceptive effects of ABX in a rat model of oxaliplatin‐induced neuropathic pain [13]. To assess the outcomes in animals, biochemical parameters were analyzed, including tumor necrosis factor‐alpha (TNF‐α), thiobarbituric acid reactive substances (TBARS), superoxide anion generation in the sciatic nerve, and myeloperoxidase (MPO) activity. Additionally, behavioral tests were conducted to evaluate nociception: the hot plate test (thermal hyperalgesia of the paw), the acetone drop test (cold allodynia of the paw), and the tail immersion test in cold water (cold hyperalgesia of the tail) [23, 24, 25].

Thus, the study observed that pharmacological co‐treatment with ABX effectively improved oxaliplatin‐induced neuropathic pain, as ABX significantly alleviated hyperalgesia and pain‐related allodynia behaviors in rats. This finding is supported by evidence demonstrating that ABX possesses antinociceptive, antioxidant, and anti‐inflammatory properties [10, 26]. The neuroprotective and antinociceptive potential of ABX can be attributed to the reduction of oxidative stress markers, inflammatory mediators, and the inhibition of voltage‐gated sodium channels in chemotherapy‐induced peripheral neuropathic pain [13].

Other studies have also evaluated the antinociceptive effect of ABX, such as the study by Hama et al. in 2010 [11], which investigated this effect in rats with spinal cord injury‐induced neuropathic pain. Similar to other studies, the antinociceptive effect was assessed through behavioral tests, including the mechanical hypersensitivity test and the heat hypersensitivity test [27, 28].

The results regarding the antinociceptive effect of ABX showed that the highest dose (1000 mg/kg) significantly reversed mechanical hypersensitivity, starting 30 min after administration and persisting for at least 3 h (*p* < 0.05). Regarding heat withdrawal latency, an increase in withdrawal latencies was observed in rats treated with 300 mg/kg of ABX, while a partial reversal of heat hypersensitivity was noted 90 min after administration of a dose of 100 mg/kg [11].

We highlight that this review is the first to comprehensively examine the activity of ABX in three distinct actions: anti‐inflammatory, analgesic, and antioxidant in experimental models of various non‐pulmonary diseases, revealing a promising therapeutic potential for managing inflammation, pain, and oxidative stress in different tissues. However, some limitations should be considered, such as the limited number of recent publications, methodological heterogeneity among the included studies, and a potential publication bias due to the small sample size in the analysis. Nevertheless, these biases were minimized through an extensive search of the international literature.

## 5 Conclusion

Although ABX has been used for decades as a mucolytic and expectorant in the treatment of respiratory diseases, few studies have explored its use for other purposes. Nonetheless, this systematic review underscores its therapeutic potential as an antioxidant, anti‐inflammatory, and antinociceptive agent, emphasizing its versatility in experimental models of diseases not related to the respiratory system.

The reviewed studies indicate that ABX exerts beneficial effects in various pathological conditions, acting as an antioxidant modulator by increasing the levels of antioxidant enzymes such as SOD, CAT, and GSH while reducing oxidative stress markers like MDA. Regarding inflammation, ABX reduced pro‐inflammatory cytokines (TNF‐α, IL‐1β, IL‐6) and increased the expression of the anti‐inflammatory cytokine IL‐10. It also prevented histological damage, edema, and inflammation and contributed to maintaining redox balance. As for its antinociceptive effect, ABX reduced hyperalgesia and allodynia, possibly through the inhibition of voltage‐gated sodium channels.

However, some limitations must be considered. Methodological heterogeneity, a limited number of recent studies, and the overall scarcity of publications restrict the generalization of findings and hinder the feasibility of more robust analyses, such as a meta‐analysis.

Finally, it is important to highlight the relevance of ABX as a promising therapeutic agent for the treatment of inflammatory, oxidative, and pain‐related conditions. However, further experimental research on non‐pulmonary diseases, as well as well‐designed clinical trials, is essential to validate its efficacy.

## Author Contributions

Michelline Joana Tenório Albuquerque Madruga Mesquita and João Batista Santos Garcia conceived the idea for the review, defined the guiding question and inclusion/exclusion criteria, and supervised the study. Michelline Joana Tenório Albuquerque Madruga Mesquita, Anne Caroline Silva Nogueira da Cruz, and Rafael de Abreu Lima developed the protocol, registered the review, and assisted in defining the search, selection, and data extraction methods. Michelline Joana Tenório Albuquerque Madruga Mesquita, Joana Tenório‐Meireles, Arney José Nogueira Farias, and Isabela Nogueira Santos conducted the database search, applying strategies and filters. Michelline Joana Tenório Albuquerque Madruga Mesquita and João Batista Santos Garcia reviewed the titles and abstracts for study selection. Maria do Socorro de Sousa Cartágenes assisted in screening and resolved author discrepancies. Michelline Joana Tenório Albuquerque Madruga Mesquita, Gustavo Frota, and Taciana Gabrielle Pinheiro de Moura Carvalho extracted and organized the data from the included articles. Michelline Joana Tenório Albuquerque Madruga Mesquita, Jorge Antônio Meireles‐Teixeira, and Rafael Cardoso Carvalho assessed the risk of bias, analyzed the data, and assisted in writing the results. Michelline Joana Tenório Albuquerque Madruga Mesquita, Rafael Antônio Freire Carvalho, and Tereza Prazeres reviewed the text, translated it, ensured compliance with the journal's guidelines, submitted the manuscript, and followed the editorial process.

## Funding

The authors have nothing to report.

## Ethics Statement

Ethical approval was not required for this systematic review as it did not involve human or animal subjects.

## Consent

The authors have nothing to report.

## Conflicts of Interest

The authors declare no conflicts of interest.

## Permission to Reproduce Material From Other Sources

No previously published material requiring permission for reproduction was used in this study.

## Clinical Trial Registration

The authors have nothing to report.

## Data Availability

All data generated or analyzed during this study are included in this published article and its supporting information files. Additional details can be provided upon reasonable request.
